# Presurgical selection of the ideal aneurysm clip by the use of a three-dimensional planning system

**DOI:** 10.1007/s10143-022-01794-4

**Published:** 2022-05-12

**Authors:** Eike Schwandt, Ralf Kockro, Andreas Kramer, Martin Glaser, Florian Ringel

**Affiliations:** 1 Department of Neurosurgery, University Medical Center Mainz, Langenbeckstr. 1, 55131 Mainz, Germany; 2 Department of Neurosurgery, Klinik Hirslanden, Zurich, Switzerland

**Keywords:** Cerebral aneurysm, Clipping, Simulation, Aneurysm remnant

## Abstract

Aneurysm occlusion rate after clipping is higher than after endovascular treatment. However, a certain percentage of incompletely clipped aneurysms remains. Presurgical selection of the proper aneurysm clips could potentially reduce the rate of incomplete clippings caused by inadequate clip geometry. The aim of the present study was to assess whether preoperative 3D image-based simulation allows for preoperative selection of a proper aneurysm clip for complete occlusion in individual cases. Patients harboring ruptured or unruptured cerebral aneurysms prior to surgical clipping were analyzed. CT angiography images were transferred to a 3D surgical-planning station (Dextroscope®) with imported models of 58 aneurysm clips. Intracranial vessels and aneurysms were segmented and the virtual aneurysm clips were placed at the aneurysm neck. Operating surgeons had information about the selected aneurysm clip, and patients underwent clipping. Intraoperative clip selection was documented and aneurysm occlusion rate was assessed by postoperative digital subtraction angiography. Nineteen patients were available for final analysis. In all patients, the most proximal clip at the aneurysm neck was the preselected clip. All aneurysms except one were fully occluded, as assessed by catheter angiography. One aneurysm had a small neck remnant that did not require secondary surgery and was occluded 15 months after surgery. 3D image-based preselection of a proper aneurysm clip can be translated to the operating room and avoids intraoperative clip selection. The associated occlusion rate of aneurysms is high.

## Introduction

The main aim of surgical treatment of ruptured or unruptured aneurysms is (i) complete aneurysm occlusion to avoid hemorrhage from an aneurysm residual or recurrent aneurysm and (ii) ideal patient outcome, i.e., the lowest possible treatment-associated morbidity. To achieve these aims, several technical improvements have been implemented over the last decade. These include less invasive craniotomies and retractorless approaches to reduce approach-related morbidity and augmented reality techniques to optimize the surgical approach angle and trajectory to an aneurysm [1–10]. Intraoperative methods to soften complex aneurysms, such as adenosine-induced temporary cardiac arrest or rapid cardiac pacing, allow for improved aneurysm control and clip placement [11–14]. Furthermore, microprobe Doppler sonography and especially indocyanine green (ICG) fluorescence video angiography provide for the intraoperative assessment of the completeness of aneurysm occlusion and exclusion of parent vessel blood flow reduction by occlusion or stenosis [15–19]. Finally, intraoperative electrophysiological monitoring of motor-evoked potentials allows for an online assessment of the functional integrity of motor pathways [20].

While these techniques improve several aspects of aneurysm clipping, the selection of the aneurysm clip or multiple clips with ideal geometry for occlusion of a given aneurysm remains an issue regarding the intraoperative spatial sense of the operating surgeon but is a crucial aspect of aneurysm occlusion. Therefore, preoperative selection of an ideal clip for aneurysm occlusion might improve surgical clipping.

A presurgical selection of the ideal clip could have several advantages. First, presurgical selection could identify the ideal clip for aneurysm occlusion, thereby improving occlusion and avoiding intraoperative changes of potentially suboptimal clips. Furthermore, medical device regulations increasingly prohibit clip resterilization and require single-packed sterile aneurysm clips, which impedes surgeons’ ability to try aneurysm clips of various geometries to identify the one providing the best occlusion.

Presurgical clip selection could overcome these problems. Modern 3D image-processing techniques enable a selection of the ideal clip geometry for an aneurysm prior to surgery. The present study assessed whether presurgical clip selection using a 3D planning system is feasible to choose an adequate clip for aneurysm occlusion.

## Material and methods

### Patients and evaluations

**Fig. 1:**
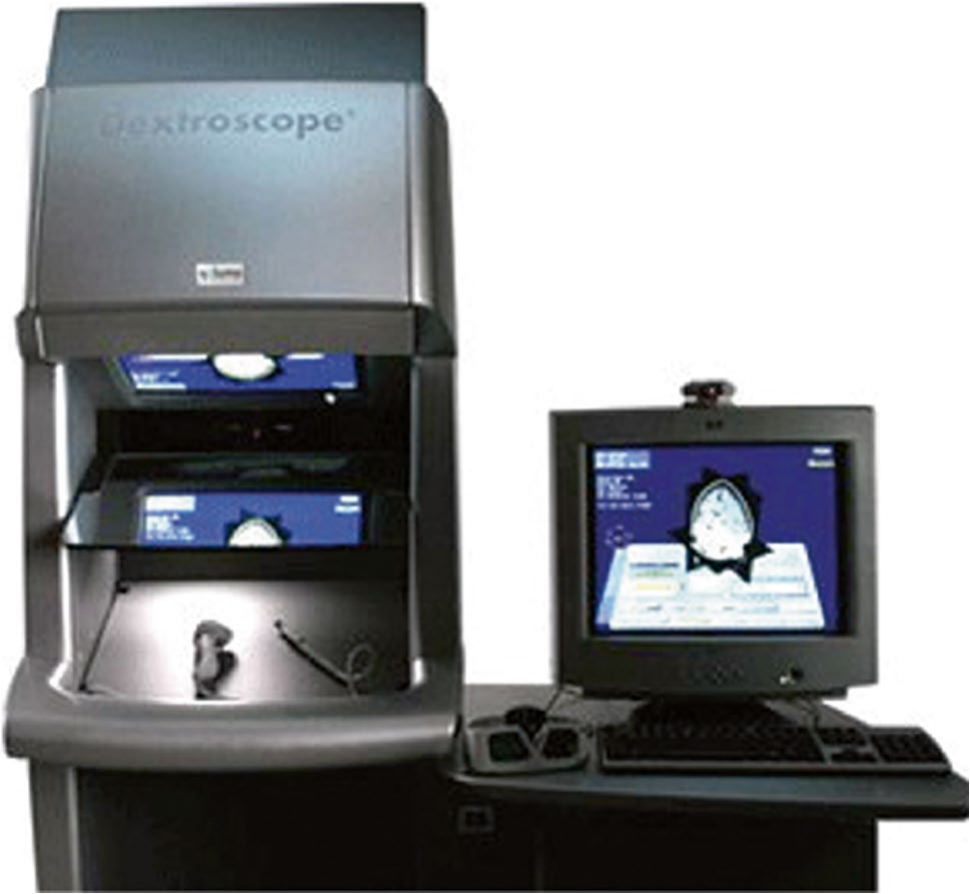
Dextroscope® 3D workstation

Over a 1-year period, patients harboring ruptured and unruptured intracranial aneurysms planned for surgical clip occlusion after interdisciplinary discussion and available for preoperative planning were assessed prospectively. Prior to surgery, patients’ routine imaging data were uploaded to a 3D planning workstation (Dextroscope®, Bracco Diagnostics, Monroe Township, USA) (Fig. 1) and the ideal clip geometry for aneurysm occlusion was assessed with the surgeon performing the clipping. The preselected clip was documented and surgical clipping performed in a routine fashion. After surgery, the clips used were compared to the preselected ones and the cases were rated as a fit (preselected aneurysm clip type used) or non-fit (preselected aneurysm clip not used). The quality of aneurysm occlusion was rated by a routinely performed postoperative digital subtraction angiography.

### Patient imaging

A computer tomography angiography (CTA) was acquired as a routine imaging diagnostic prior to surgery in all patients. Patients underwent scanning in a Toshiba Aquillion S32 CT scanner (Toshiba Medical Systems GmbH Deutschland, Neuss, Germany) to obtain 3D CTA datasets, and 75 ml of contrast agent (Imeron® 400, Bracco Imaging Deutschland GmbH, Konstanz, Germany) was injected at a rate of 3 ml/s. CT scanning was started when the contrast agent bolus was detected in the extracranial internal carotid artery. CT data were acquired with a slice thickness of 0.5 mm from the foramen magnum to the vertex.

Additionally, all but 4 patients were diagnosed with a presurgical digital subtraction angiography (DSA) (Philips Integris Allura, Philips Deutschland, Hamburg, Germany). In 10 cases of unruptured aneurysms, a magnetic resonance angiography (MRA) (Siemens MAGNETOM Skyra or MAGNETOM Trio, Siemens Healthcare GmbH, Erlangen, Germany) TOF-3D dataset with a slice thickness of 0.5 mm was available.

Postoperatively, the clipping results were evaluated by digital subtraction angiography for completeness of aneurysm occlusion and aneurysm remnants.

### Clip imaging

**Fig. 2:**
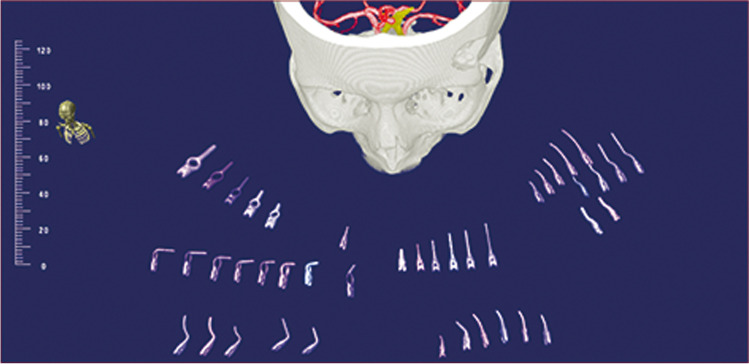
Collection of aneurysm clips imported to the Dextroscope® planning station. Each clip is an individual 3D object which is movable in all planes without any limitation. Thereby, simulation of different clipping scenarios is possible

From a set of aneurysm clips, 58 types—49 L-Clips (Peter Lazic, Tuttlingen, Germany) and nine Yasargil clips (Aesculap, B. Braun, Tuttlingen, Germany)—were scanned in a closed status using the same CT scanner mentioned above and exported as DICOM datasets. CT data was acquired with a thickness of 0.5 mm (X-ray tube current 150mAs, KVP 120)(Fig. 2).

### 3D surgical planning and clip preselection

Surgical 3D planning was carried out with the Dextroscope®, a stereoscopic visualization and planning workstation described previously [21]. The Dextroscope® 3D-planning station (Volume Interactions Ltd., Singapore) is a virtual reality environment in which the operator reaches with both hands behind a mirror into a computer-generated stereoscopic 3D object projection to move and manipulate objects in real-time with natural 3D hand movements. Software tools for data loading, 3D reconstruction, segmentation, thresholding, coloring, and transparency modulation are available in the system. Manual segmentation tools allow the 3D delineation of structures that cannot be segmented on the basis of grayscale thresholds.

**Fig. 3:**
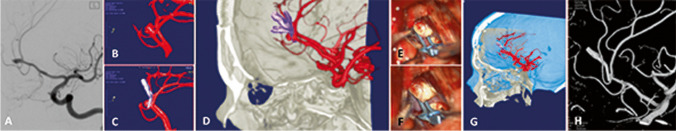
Illustrative case showing **A** a pericallosal artery aneurysm in DSA and **B** CTA segmented in the planning station. **C** and **D** Show the preoperative virtual selection of an adequate clip, **E** and **F** the intraoperative placement of the clip at the aneurysm neck, **G** and **H** the postoperative imaging result

After uploading patient CTA and where available MRA imaging data and the volumetric representations of the virtual clip collection as DICOM data, 3D volume rendering was followed by data co-registration (CTA and MRA) and fusion. The arteries and the bone of the skull were segmented as single objects and colored. The aneurysms and parent vessels were segmented from CTA data, and the closed aneurysm clips were virtually positioned over the aneurysm necks (Fig. 3). Various clipping scenarios were assessed using the clip collection. However, neither the opening nor closing of the clips nor tissue deformation could be simulated. The most suitable clip for aneurysm size, shape, and approach in virtual reality was chosen.

Additionally, voxel-editing tools allowed for a simulated craniotomy. A computer-generated crosswire simulated the focal point of a microscope and a magnification tool simulating the microscopic view into the surgical field for further planning.

### Aneurysm treatment and postoperative imaging control

The type of preselected clip was recommended to the operating surgeon prior to the surgical case. Aneurysm clipping was performed in a standard microsurgical fashion, using intraoperative micro-Doppler analysis and ICG angiography. The type and number of clips used intraoperatively for aneurysm occlusion were documented. All patients underwent routine postoperative digital subtraction angiography for assessment of aneurysm occlusion and parent vessel patency. Aneurysm occlusion was classified according to a modified Montreal scale in classes I–III, where class I is a complete occlusion, class II a neck remnant, and class III an aneurysm remnant [22]. Any clip-associated parent vessel stenosis was documented.

## Results

**Fig. 4:**
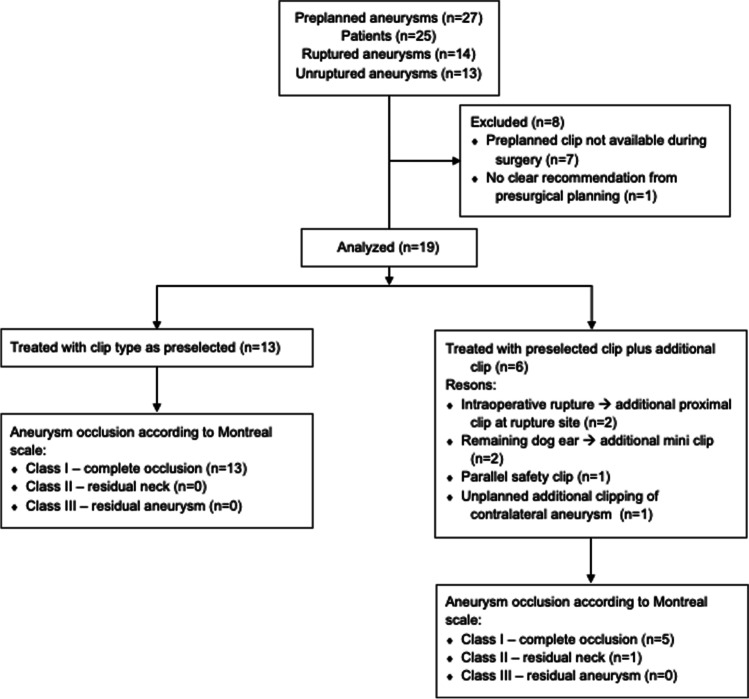
Flow chart of patients

Figure 4 summarizes all relevant results.

### Patients, aneurysms, clip preselection

Twenty-five patients harboring 27 intracranial aneurysms were analyzed. Fourteen patients had subarachnoid hemorrhages from ruptured aneurysms; two of those patients carried another unruptured aneurysm, and 11 additional patients carried unruptured aneurysms. Aneurysm locations were the internal carotid artery (ICA; *n* = 2), anterior communicating artery (AComA; *n* = 7), anterior cerebral artery (ACA; *n* = 1), pericallosal artery (PA; *n* = 4), middle cerebral artery (MCA; *n* = 10), posterior communicating artery (PComA; *n* = 2), and tip of the basilar artery (BA; *n* = 1). All aneurysms were analyzed for the ideal clip prior to surgical treatment. For a single complex MCA aneurysm, no clear recommendation could be generated from the presurgical analysis. Clips were preselected for all remaining aneurysms. Three-dimensional planning did not change the treatment decision for clipping in any case. During surgery, the preselected clips were not available in seven cases. Therefore, the 19 remaining aneurysms, characterized in Table 1, were analyzed.

**Table 1:** Aneurysm characteristics

Patient	Age [yrs]	SAH [y|n]	Aneurysm location	Dome projection	Max. dia-meter [mm]	Neck width [mm]	Neck/dome ratio	Clips applied [n] /reason ad. clip	An. remnant [y|n, projection]
1	50	n	BA tip	Rostral	3.3	2.3	1.44	1	n
2	73	n	AComA	Rostral	5.8	4.8	1.21	2/safety	y, posterobasal
3	35	n	MCA left	Lateral	4.7	4.5	1.07	1	n
4	56	n	MCA left	Lateral	7.9	5.9	1.34	2/dog ear	n
5	52	n	ICA left	Dorsal	9.5	3.8	2.5	2/dog ear	n
6	47	n	PComA right	Ventrocaudal	4	2.5	1.57	2/2^nd^ aneurysm	n
7	58	n	ICA left	Dorsal	2.8	2	1.40	1	n
8	42	n	PA right	Caudolateral	3.8	2.8	1.36	1	n
9	65	n	PA left	Rostral	2.8	1.5	1.87	1	n
10	72	n	MCA right	Lateral	11.6	2.6	4.46	1	n
11	50	n	MCA left	Lateral	4.3	2.1	2.05	1	n
12	34	y	AComA	Lateral	6.6	3.2	2.06	2/rupture	n
13	57	y	MCA left	Dorsolateral	3.7	1.7	2.18	1	n
14	67	y	AComA	Ventrocaudal	7.5	2.1	3.57	1	n
15	41	y	MCA left	Lateral	7.1	3.6	1.97	1	n
16	30	y	ACA left	Rostrocaudal	5.3	2.3	2.3	1	n
17	44	y	PA left	Rostral	5	2.6	1.92	1	n
18	57	y	PComA right	Dorsolateral	14.1	4.6	3.06	1	n
19	77	y	AComA	Rostral	5.5	2.7	2.04	2/rupture	n

*BA*, basilary artery; *AComA*, anterior communicating artery; *MCA*, middle cerebral artery; *ICA*, internal carotid artery; *PComA*, posterior communicating artery; *PA*, pericallosal artery; *ad*., additional; *an.*, aneurysm; *y*, yes; *n*, no

### Aneurysm clipping

The preselected clips were used in all analyzed cases. Thirteen of 19 aneurysms required a solitary clip for aneurysm occlusion. For the remaining six, the preselected clip was used together with additional clips. In two of those cases, an intraoperative aneurysm rupture occurred, and clipping of the rupture site was necessary. However, in those cases, the preselected clip was used as most proximal clip at the aneurysm neck in addition to the more distal clips at the rupture site. In another two cases, a mini clip was used in addition to the preselected clip to occlude a small remaining dog ear. Concerning other cases in which additional clips were used, in one, a second clip was placed parallel but distal to the preselected clip as a safety measure, as the occlusion of clip branches of the first clip was interpreted as incomplete as a consequence of aneurysm wall rigidity. In the remaining case, an additional unplanned aneurysm was clipped.

Other than four intraoperative aneurysm ruptures, no further intraoperative complications occurred.

### Aneurysm occlusion and parent vessel patency

All cases underwent postoperative digital subtraction angiography. Aneurysm occlusion was classified as Montreal class I, II, and III in 18, 1, and 0 aneurysms, respectively (Fig. 4). Thereby, the rate of incomplete occlusions with aneurysm remnants was 5%. No clip-associated parent vessel occlusion or stenosis was detected in any postoperative imaging. The persisting small dog ear in one case was followed up and occluded spontaneously, as detected during a routine follow-up digital subtraction angiography at 15 months after surgery.

## Discussion

The current study analyzed the feasibility of presurgical selection of an adequate clip for occlusion of a cerebral aneurysm with the use of a 3D planning system. The results show that presurgical clip selection could be translated to the operating room in all cases, i.e., preselected clips were used as the most proximal clip at the aneurysm neck. Aneurysm occlusion was complete in all but one case, as evaluated by postoperative angiography.

While the rate of aneurysm remnants after microsurgical clipping of cerebral aneurysms is lower than after endovascular treatment, a certain rate of incomplete clipping remains. These aneurysm remnants are associated with a risk of rupture or ameurysm regrowth, and retreatment is necessary in a subgroup of patients. The rate of incomplete clippings ranges between 1.6 and 42%, and certain risk factors for incomplete clipping have been identified as (i) aneurysm location and an (ii) aneurysm size above 12 mm [23–29]. Microsurgical clipping techniques including bipolar remodeling target to reduce aneurysm remnants [30]. Intraoperative techniques such as ICG videoangiography or catheter angiography aim to immediately assess aneurysm occlusion and allow for clip correction and improvement of occlusion [16, 31]. In addition to intraoperative assessment, presurgical planning and simulation could improve aneurysm occlusion, and various approaches are in use[32].

Different approaches have been used to simulate aneurysm clipping using virtual or physical models. Futami et al. tried to simulate aneurysm clipping with a single straight clip and assessed the associated risk of an aneurysm remnant [33]. Using 3D CT angiography, a virtual intraoperative view was simulated and a straight line was traced along the aneurysm neck to simulate clipping. Subsequently, images were rotated and aneurysm remnants identified in 42% of virtual cases; a strong correlation between predicted remnants and intraoperative remnants was visualized by endoscopy. Wong et al. used the same approach as the present study (i.e., presurgical Dextroscope® planning) but did not assess whether the planned virtual clips could be translated to intraoperative situations and lead to sufficient aneurysm occlusion [34]. Our present series shows that virtual selection of a proper clip translates to intraoperative situations and potentially improves aneurysm occlusion. A deficit of this virtual 3D imaging-based approach is the missing simulation of vessel and aneurysm deformation after clip application. While a deformation simulation would be desirable, it is not necessarily required for an adequate simulation, as shown by our series.

A further approach to presurgical clipping simulation taking aneurysm deformation into account is the use of 3D-printed aneurysm models prior to surgery [35–44]. These printed models represent an attempt to simulate aneurysm clipping with increasing complexity, adding blood flow simulation and aneurysm pulsation in some studies. These models fulfil two purposes as follows: (i) individual case-based planning and (ii) simulation for overall surgical training of aneurysm clipping.

However, while aneurysm wall deformation can certainly be simulated by complex models, the quality of deformation simulation by artificial materials remains unclear. The disadvantages of these 3D-printed models are the time and costs of preparation, reducing their feasibility for individual presurgical planning applications, especially in emergency situations of ruptured aneurysms. Therefore, these models certainly carry a high potential for overall surgeon training in aneurysm clipping but are less useful for individual planning.

Furthermore, our concept of 3D virtual planning does not allow to include aneurysm reconstruction techniques as bipolar remodeling [30] as any changes in configuration of the aneurysm cannot be simulated prior to surgery. The intraoperative rupture of an aneurysm can be associated with an unplanned change in configuration; however, the location for clip application, i.e. the aneurysm neck usually remains unchanged by a rupture of the dome, and therefore, presurgical planning for a neck clip remains valid.

The advantage of our present approach is the ease of use, the use of available imaging information for most cases (avoiding additional imaging), and the availability as an ad hoc planning method without costly or time-consuming preparation.

A limitation of the present study is the rather small number of aneurysms analyzed after presurgical 3D planning. Furthermore, most aneurysms assessed are located in the anterior circulation and complex posterior circulation aneurysms where presurgical 3D clip preselection might be even more helpful are not sufficiently presented.

In conclusion, 3D virtual preselection of proper aneurysm clips allows a clip geometry to be selected for the occlusion of individual cerebral aneurysms. This clip selection can be translated to the operating room, leading to a high rate of cerebral aneurysm occlusion without remnants. The system does not require time-consuming additional preparation and is useful even in emergency situations of ruptured cerebral aneurysms.

## Data Availability

Original data is available via the authors; commercial material availability is marked in the text.

## References

[1] Mori K, Wada K, Otani N, Tomiyama A, Toyooka T, Takeuchi S, Yamamoto T, Nakao Y, Arai H (2018) Keyhole strategy aiming at minimizing hospital stay for surgical clipping of unruptured middle cerebral artery aneurysms. J Neurosurg:1–8. 10.3171/2017.10.JNS171973 10.3171/2017.10.JNS171973 29676694

[2] Raabe A, Beck J, Rohde S, Berkefeld J, Seifert V (2006). Three-dimensional rotational angiography guidance for aneurysm surgery. J Neurosurg.

[3] Stidd DA, Wewel J, Ghods AJ, Munich S, Serici A, Keigher KM, Theessen H, Moftakhar R, Lopes DK (2014). Frameless neuronavigation based only on 3D digital subtraction angiography using surface-based facial registration. J Neurosurg.

[4] Sun H, Safavi-Abbasi S, Spetzler RF (2016). Retractorless surgery for intracranial aneurysms. J Neurosurg Sci.

[5] Toyooka T, Otani N, Wada K, Tomiyama A, Takeuchi S, Fujii K, Kumagai K, Fujii T, Mori K (2018). Head-up display may facilitate safe keyhole surgery for cerebral aneurysm clipping. J Neurosurg.

[6] Tra H, Huynh T, Nguyen B (2018). Minipterional and supraorbital keyhole craniotomies for ruptured anterior circulation aneurysms: experience at single center. World Neurosurg.

[7] Vassallo R, Kasuya H, Lo BWY, Peters T, Xiao Y (2018). Augmented reality guidance in cerebrovascular surgery using microscopic video enhancement. Healthc Technol Lett.

[8] Wada K, Nawashiro H, Ohkawa H, Arimoto H, Takeuchi S, Mori K (2015). Feasibility of the combination of 3D CTA and 2D CT imaging guidance for clipping microsurgery of anterior communicating artery aneurysm. Br J Neurosurg.

[9] Wong JH, Tymianski R, Radovanovic I, Tymianski M (2015). Minimally invasive microsurgery for cerebral aneurysms. Stroke.

[10] Yu LH, Yao PS, Zheng SF, Kang DZ (2015). Retractorless surgery for anterior circulation aneurysms via a pterional keyhole approach. World Neurosurg.

[11] Konczalla J, Platz J, Fichtlscherer S, Mutlak H, Strouhal U, Seifert V (2018). Rapid ventricular pacing for clip reconstruction of complex unruptured intracranial aneurysms: results of an interdisciplinary prospective trial. J Neurosurg.

[12] Luostarinen T, Takala RS, Niemi TT, Katila AJ, Niemela M, Hernesniemi J, Randell T (2010) Adenosine-induced cardiac arrest during intraoperative cerebral aneurysm rupture. **World Neurosurg 73:**79–83; discussion e79. 10.1016/j.surneu.2009.06.018 10.1016/j.surneu.2009.06.018 20860932

[13] Vealey R, Koht A, Bendok BR (2017). Multidose adenosine used to facilitate microsurgical clipping of a cerebral aneurysm complicated by intraoperative rupture: a case report. A A Case Rep.

[14] Zall S, Eden E, Winso I, Volkmann R, Sollevi A, Ricksten SE (1990). Controlled hypotension with adenosine or sodium nitroprusside during cerebral aneurysm surgery: effects on renal hemodynamics, excretory function, and renin release. Anesth Analg.

[15] Pereira BJ, Holanda VM, Giudicissi-Filho M, Borba LA, de Holanda CV, de Oliveira JG (2015). Assessment of cerebral blood flow with micro-doppler vascular reduces the risk of ischemic stroke during the clipping of intracranial aneurysms. World Neurosurg.

[16] Raabe A, Beck J, Gerlach R, Zimmermann M, Seifert V (2003) Near-infrared indocyanine green video angiography: a new method for intraoperative assessment of vascular flow. **Neurosurgery 52:**132–139; discussion 139. 10.1097/00006123-200301000-00017 10.1097/00006123-200301000-00017 12493110

[17] Raabe A, Beck J, Seifert V (2005) Technique and image quality of intraoperative indocyanine green angiography during aneurysm surgery using surgical microscope integrated near-infrared video technology. Zentralbl Neurochir 66**:**1–6; discussion 7–8. 10.1055/s-2004-836223 10.1055/s-2004-836223 15744621

[18] Raabe A, Nakaji P, Beck J, Kim LJ, Hsu FP, Kamerman JD, Seifert V, Spetzler RF (2005). Prospective evaluation of surgical microscope-integrated intraoperative near-infrared indocyanine green videoangiography during aneurysm surgery. J Neurosurg.

[19] Riva M, Amin-Hanjani S, Giussani C, De Witte O, Bruneau M (2018). Indocyanine green videoangiography in aneurysm surgery: systematic review and meta-analysis. Neurosurgery.

[20] Thomas B, Guo D (2017). The diagnostic accuracy of evoked potential monitoring techniques during intracranial aneurysm surgery for predicting postoperative ischemic damage: a systematic review and meta-analysis. World Neurosurg.

[21] Kockro RA, Serra L, Tseng-Tsai Y, Chan C, Yih-Yian S, Gim-Guan C, Lee E, Hoe LY, Hern N, Nowinski WL (2000) Planning and simulation of neurosurgery in a virtual reality environment. Neurosurgery 46:118–135; discussion 135–117. 10626943

[22] Raymond J, Guilbert F, Weill A, Georganos SA, Juravsky L, Lambert A, Lamoureux J, Chagnon M, Roy D (2003). Long-term angiographic recurrences after selective endovascular treatment of aneurysms with detachable coils. Stroke.

[23] Ahn SS, Kim YD (2010). Three-dimensional digital subtraction angiographic evaluation of aneurysm remnants after clip placement. J Korean Neurosurg Soc.

[24] Ihm EH, Hong CK, Shim YS, Jung JY, Joo JY, Park SW (2010). Characteristics and management of residual or slowly recurred intracranial aneurysms. J Korean Neurosurg Soc.

[25] Jabbarli R, Pierscianek D, Wrede K, Dammann P, Schlamann M, Forsting M, Muller O, Sure U (2016). Aneurysm remnant after clipping: the risks and consequences. J Neurosurg.

[26] Kang HS, Han MH, Kwon BJ, Jung SI, Oh CW, Han DH, Chang KH (2004). Postoperative 3D angiography in intracranial aneurysms. AJNR Am J Neuroradiol.

[27] Rauzzino MJ, Quinn CM, Fisher WS, 3rd (1998) Angiography after aneurysm surgery: indications for "selective" angiography. **Surg Neurol 49:**32–40; discussion 40–31. 10.1016/s0090-3019(97)00035-9 10.1016/s0090-3019(97)00035-9 9428892

[28] Sindou M, Acevedo JC, Turjman F (1998). Aneurysmal remnants after microsurgical clipping: classification and results from a prospective angiographic study (in a consecutive series of 305 operated intracranial aneurysms). Acta Neurochir (Wien).

[29] Thornton J, Bashir Q, Aletich VA, Debrun GM, Ausman JI, Charbel FT (2000) What percentage of surgically clipped intracranial aneurysms have residual necks? **Neurosurgery 46:**1294–1298; discussion 1298–1300. 10.1097/00006123-200006000-00003 10.1097/00006123-200006000-00003 10834634

[30] Choque-Velasquez J, Hernesniemi J (2018). Microsurgical clipping of a large ruptured anterior communicating artery aneurysm. Surg Neurol Int.

[31] Westermaier T, Linsenmann T, Homola GA, Loehr M, Stetter C, Willner N, Ernestus RI, Solymosi L, Vince GH (2016). 3D rotational fluoroscopy for intraoperative clip control in patients with intracranial aneurysms–assessment of feasibility and image quality. BMC Med Imaging.

[32] Marinho P, Thines L, Verscheure L, Mordon S, Lejeune JP, Vermandel M (2012). Recent advances in cerebrovascular simulation and neuronavigation for the optimization of intracranial aneurysm clipping. Comput Aided Surg.

[33] Futami K, Nakada M, Iwato M, Kita D, Miyamori T, Yamashita J (2004) Simulation of clipping position for cerebral aneurysms using three-dimensional computed tomography angiography. Neurol Med Chir (Tokyo) 44:6–12; discussion 13. 10.2176/nmc.44.6 10.2176/nmc.44.6 14959930

[34] Wong GK, Zhu CX, Ahuja AT, Poon WS (2007) Craniotomy and clipping of intracranial aneurysm in a stereoscopic virtual reality environment. **Neurosurgery 61:**564–568; discussion 568–569. 10.1227/01.NEU.0000290904.46061.0D 10.1227/01.NEU.0000290904.46061.0D 17881970

[35] Benet A, Plata-Bello J, Abla AA, Acevedo-Bolton G, Saloner D, Lawton MT (2015). Implantation of 3D-printed patient-specific aneurysm models into cadaveric specimens: a new training paradigm to allow for improvements in cerebrovascular surgery and research. Biomed Res Int.

[36] Gallardo FC, Bustamante JL, Martin C, Orellana CM, Rojas Caviglia M, Garcia Oriola G, Diaz AI, Rubino PA, Quilis Quesada V (2020). Novel simulation model with pulsatile flow system for microvascular training, research, and improving patient surgical outcomes. World Neurosurg.

[37] Joseph FJ, Weber S, Raabe A, Bervini D (2020). Neurosurgical simulator for training aneurysm microsurgery-a user suitability study involving neurosurgeons and residents. Acta Neurochir (Wien).

[38] Lan Q, Zhu Q, Xu L, Xu T (2020). Application of 3D-printed craniocerebral model in simulated surgery for complex intracranial lesions. World Neurosurg.

[39] Leal A, Souza M, Nohama P (2019). Additive manufacturing of 3D biomodels as adjuvant in intracranial aneurysm clipping. Artif Organs.

[40] Leal AG, Mori YT, Nohama P, de Souza MA (2019). Three-dimensional hollow elastic models for intracranial aneurysm clipping election - a case study. Annu Int Conf IEEE Eng Med Biol Soc.

[41] Liu Y, Gao Q, Du S, Chen Z, Fu J, Chen B, Liu Z, He Y (2017). Fabrication of cerebral aneurysm simulator with a desktop 3D printer. Sci Rep.

[42] Mashiko T, Kaneko N, Konno T, Otani K, Nagayama R, Watanabe E (2017). Training in cerebral aneurysm clipping using self-made 3-dimensional models. J Surg Educ.

[43] Ryan JR, Almefty KK, Nakaji P, Frakes DH (2016). Cerebral aneurysm clipping surgery simulation using patient-specific 3D printing and silicone casting. World Neurosurg.

[44] Wang L, Ye X, Hao Q, Chen Y, Chen X, Wang H, Wang R, Zhao Y, Zhao J (2017). Comparison of two three-dimensional printed models of complex intracranial aneurysms for surgical simulation. World Neurosurg.

